# Effect of Da-Cheng-Qi decoction for treatment of acute kidney injury in rats with severe acute pancreatitis

**DOI:** 10.1186/s13020-018-0195-8

**Published:** 2018-07-13

**Authors:** Ling Yuan, Lv Zhu, Yumei Zhang, Huan Chen, Hongxin Kang, Juan Li, Xianlin Zhao, Meihua Wan, Yifan Miao, Wenfu Tang

**Affiliations:** Department of Integrative Medicine, West China Hospital, Sichuan University, Chengdu, 610041 Sichuan People’s Republic of China

**Keywords:** Da-Cheng-Qi decoction, Acute pancreatitis, Acute kidney injury, Tissue distribution, Inflammatory response

## Abstract

**Background:**

The traditional Chinese formula Da-Cheng-Qi-decoction (DCQD) has been used to treat acute pancreatitis for decades. DCQD could ameliorate the disease severity and the complications of organ injuries, including those of the liver and lungs. However, the pharmacological effects in the kidney, a target organ, are still unclear. This study aimed to investigate the herbal tissue pharmacology of DCQD for acute kidney injury (AKI) in rats with severe acute pancreatitis (SAP).

**Methods:**

Rats were randomly divided into the sham-operation group (SG), the model group (MG) and the low-, medium- and high-dose treatment groups (LDG, MDG, and HDG, respectively). Sodium taurocholate (3.5%) was retrogradely perfused into the biliopancreatic duct to establish the model of SAP in rats. Different doses of DCQD were administered to the treatment groups 2 h after the induction of SAP. The major components of DCQD in kidney tissues were detected by HPLC–MS/MS. Inflammatory mediators in the kidney tissues, as well as serum creatinine (Scr), blood urea nitrogen (BUN) and pathologic scores, were also evaluated.

**Results:**

Ten components of DCQD were detected in the kidneys of the treatment groups, and their concentrations increased dose-dependently. Compared with the SG, the levels of inflammatory mediators, Scr, BUN and pathological scores in the MG were obviously increased (*p* < 0.05). The high dose of DCQD showed a maximal effect in downregulating the pro-inflammatory mediators interleukin-6 (IL)-6 and tumour necrosis factor-α (TNF-α), upregulating anti-inflammatory mediators IL-4 and IL-10 in the kidney and alleviating the pathological damages. DCQD decreased the pancreas and kidney pathological scores of rats with SAP, especially in the HDG (*p* < 0.05). Compared with the MG, the level of Scr in the HDG was significantly decreased (p < 0.05).

**Conclusions:**

DCQD ameliorated AKI in rats with SAP via regulating the inflammatory response, which might be closely related to the distribution of its components in the kidney.

**Electronic supplementary material:**

The online version of this article (10.1186/s13020-018-0195-8) contains supplementary material, which is available to authorized users.

## Background

Acute pancreatitis (AP) is commonly a self-limited inflammatory disease caused by abnormally activated pancreatic digestive enzymes [1]. However, approximately 20% of AP cases develop into severe acute pancreatitis (SAP), with high mortality characterized by systemic inflammatory response syndrome (SIRS) and multiple organ injuries and even failure at an early stage [2, 3], including acute respiratory distress syndrome, acute kidney injury (AKI) and acute liver injury. AKI is diagnosed by accumulations of serum creatinine (Scr) and blood urea nitrogen (BUN) or decreased urine output, which reflects a rapid loss of the kidney’s excretory function [4]. AKI is one of the most common complications of SAP increasing the disease mortality [5, 6]. A retrospective and multi-centre study showed that nearly 69.3% of SAP patients developed AKI [5]. Acute renal failure used to be defined as the severe form of AKI [7], which leads to a drastic increase in the mortality of SAP [8–10]. Therefore, it is essential to ameliorate AKI with SAP as early as possible to decrease the mortality.

The mechanism of SAP resulting in AKI is complex. Available studies revealed that SAP-induced AKI is mainly related to SIRS [9], which involves various cytokines and inflammatory mediators, such as nuclear factor kappa B (NF-B), tumour necrosis factor (TNF)-α, interleukin (IL)-1β, IL-6, IL-10, and high mobility group box protein 1(HMGB1) [11]. Endotoxins, reactive oxygen species (ROS), phospholipase A_2_ (PLA_2_), hypoxemia, as well as a decrease in renal perfusion pressure due to abdominal compartment syndrome and an impairment of renal microcirculation for the release of pancreatic amylase, may also play important roles in the pathophysiology of SAP-induced AKI [12]. The mortality of SAP patients with AKI remains high regardless of the progress in intensive care treatment [9]. Although many worldwide guidelines have been established for AP [13, 14], there is still no exact drug protocol recommended [15] other than renal replacement therapy (RRT) for AKI with SAP [4, 9]. However, the application of RRT remains controversial in many aspects and has many potential complications [16]. A multicentre, multinational, prospective study showed that RRT practice did not align with the best evidence, and variations in practice may be responsible for substantial morbidity [17]. Hence, it is worthwhile to find new interventions for SAP-induced AKI and to explore the potential mechanism.

Da-Cheng-Qi decoction (DCQD), which is composed of *Rheum palmatum* L. (Dahuang), *Magnolia henryi* Dunn. (Houpu), *Citrus aurantium* L. (Zhishi) and *Natrii Sulphas* (Mangxiao), has been applied to treat AP effectively for decades in China [18]. We hypothesized that the concentration and distribution of the components of the herbal prescription in the target organs were related to its pharmacological effect. Based on this hypothesis, our previous studies have verified that DCQD could alleviate the injuries of the pancreas, lungs, liver and intestines by inhibiting the inflammatory response in rats with AP based on the distribution of its components in target tissues [19–22]. However, the pharmacological effects of DCQD in the kidney, a target organ, are still unclear. Herein, this study investigates the herbal tissue pharmacology of DCQD in the kidneys of rats with SAP after the administration of different doses of DCQD and explores the underlying mechanisms.

## Methods

### Information of experimental design and resources

The information regarding the experimental design, statistics, and resources used in this study are attached in the minimum standards of reporting checklist (Additional file 1).

### Animals

Forty healthy, clean grade, male Sprague–Dawley rats (SD, 220 ± 15 g) were purchased from Chengdu Dashuo Bio-Technique Co. Ltd. (Chengdu, China). The experimental protocol was performed according to the Animal Ethics Committee Guidelines of our hospital (2016001A, Chengdu, China). One week after acclimation, the animals were fasted with free access to water for 24 h prior to the experiment.

### Preparation of DCQD

The spray-dried drug powders were obtained from Chengdu Green Herbal Pharmaceutical Co. Ltd. (Chengdu, China). The processing procedure of the crude formula components has been previously described [22]. According to the *Treaties on Exogenous Febrile Disease*, the suggested dose of DCQD for a person weighting 60 kg is 57 g, composed of 12 g of Dahuang, 24 g of Houpu, 12 g of Zhishi and 9 g of Mangxiao. As we have mentioned [22], we chose 6 g/kg BW (0.6 g/100 g) as the lowest dose. The drug powders mixed at the ratio of 12:24:12:9, were reconstituted with sterile distilled water at different concentrations (0.6, 1.2 and 2.4 g/mL).

### Induction of SAP and intervention

The rats were randomly divided into five groups and marked as the low-dose treatment group (LDG, 6 g/kg BW), the medium-dose treatment group (MDG, 12 g/kg BW), the high-dose treatment group (HDG, 24 g/kg BW), the sham-operation group (SG) and the model group (MG). The SAP model was induced via biliopancreatic duct injection of 3.5% sodium taurocholate (Sigma, St. Louis, MO, USA) with a micro-infusion pump at a rate of 0.2 mL/min (1 mL/kg body weight) [20]. The rats in the SG received saline instead of 3.5% sodium taurocholate. Two hours after the operation, the rats in the treatment groups were administered DCQD intragastrically at 1 mL/100 g BW, with 0.6 g/mL for the LDG, 1.2 g/mL for the MDG and 2.4 g/mL for the HDG while the rats in the SG and MG were administered an equal volume of saline.

### Sample collections and measurements

Twenty-four hours after administration, all the rats were sacrificed, and arterial blood as well as kidney pancreas tissues were collected for measurement. Pancreas and kidney tissues were cut into slices and were fixed with 10% neutral formalin, embedded in paraffin, cut into sheets (at thickness of 5–7 μm) and then stained with haematoxylin and eosin. Pathological scores were blindly evaluated by two independent pathologists with a previously established scoring system [20, 22, 23]. The severity of edema, neutrophil infiltration, necrosis, and haemorrhage was represented on a scale of 0–4 (0 = 0%, none; 1 = 25%, mild; 2 = 26–50%, moderate; 3 = 51–75%, sever; 4 = 76%, severe).

Additional pancreas and kidney tissues were stored at − 80 °C. High-performance liquid chromatography–tandem mass spectrometry (HPLC–MS/MS) was used to measure the major components of DCQD (emodin, rhein, aloe-emodin, chrysophanol, rheochrysidin, hesperidin, naringin, naringenin, magnolol and honokiol) in the kidney tissue homogenates (10%) [24]. As we detected previously, the average contents of rhein, emodin, aloe-emodin, chrysophanol, rheochrysidin, naringin, naringenin, hesperidin, magnolol, and honokiol in DCQD were 0.86, 2.48, 1.73, 0.55, 2.61, 3.83, 4.16, 11.06, 1.11, and 1.26 mg/g respectively [24].

The levels of IL-6, TNF-a, IL-10 and IL-4 in kidney tissue homogenate were measured by using the Milliplex MAP Rat Cytokine/Chemokine magnetic bead immunoassay kit (Millipore Corporation, Billerica, MA). The blood samples were centrifuged at 3000 rmp for 5 min and the serum was distracted for Scr and BUN. The concentrations of Scr and BUN were detected by an Automatic Biochemical Analyser (AU5400, SIEMENS, Munich, Germany).

### Preparation of standard and quality control samples

The ten major components of DCQD from the previous study [21, 24] were detected in this study by HPLC–MS/MS. Quality control samples were prepared to obtain the following plasma concentrations: 120, 20, 5 and 1.25 ng/mL for rheochrysidin; 100, 25 and 6.25 ng/mL for emodin; 3750, 625, 156.25 and 39.06 ng/mL for rhein; and 600, 100, 25 and 6.25 ng/mL for aloe-emodin, naringin, chrysophanol, hesperidin, magnolol, naringenin, and honokiol. The spiked plasma samples (standard and quality control) were pretreated and detected in each analytical batch along with the unknown samples [24]. The remainder of the detected DCQD was deposited in the Public Experiment Platform of our hospital (Chengdu, China).

Data collection, peak integration, and calibration were all calculated with Analyst 1.4.2 software. Calibration curves were plotted according to the peak ratio of analytes to internal standards (ibuprofen), and the linear regression between tissue concentrations and peak area ratios were determined by 1/χ^2^. Concentrations of QC and unknown samples were measured by interpolation from the calibration curves [24].

### Statistical analysis

The statistical analysis was performed with PEMS3.1 for Windows (Sichuan University, China). All the data were expressed as the mean ± standard deviation (mean ± SD). One-way repeated-measures ANOVA, followed by multiple pair-wise comparisons using the Student–Neuman–Keuls procedure, was applied to the analysis of multiple groups. The data was considered significantly different when *p* < 0.05.

## Results

### Ten components of DCQD detected in kidney tissues

**Fig. 1:**
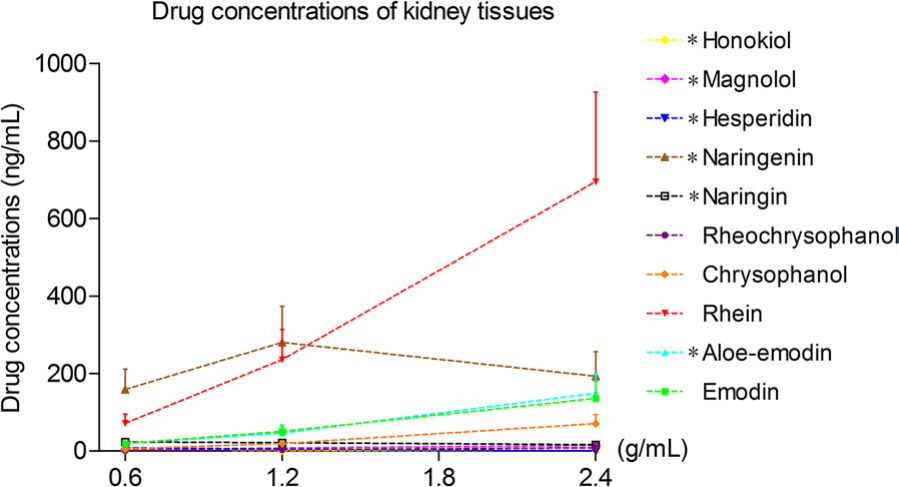
Kidney distribution of the ten major absorbed components from DCQD in rats with SAP. Rats (n = 8 per group) were orally administered different dosages of DCQD (0.6 g/mL for the low-dose group, 1.2 g/mL for the medium-dose group, and 2.4 g/mL for the high-dose group by body weight) 2 h after operation. After 24 h, the kidney tissues were collected to determine the concentrations of the components from DCQD using a sensitive HPLC–MS/MS method. The results are expressed as the mean ± SD

The ten major components of DCQD were all detected in the kidney tissues. The concentrations of emodin, rhein, aloe-emodin, chrysophanol, rheochrysidin and magnolol increased with the DCQD dose and showed significant differences when compared to the other treatment groups. The concentrations of hesperidin, naringin, naringenin, and honokiol were not as closely related to the dose. Rhein and naringenin were relatively higher than the other compounds of DCQD in all the treatment groups (Fig. 1).

### DCQD downregulated the pro-inflammatory mediators and upregulated the anti-inflammatory mediators in kidney tissues

**Fig. 2:**
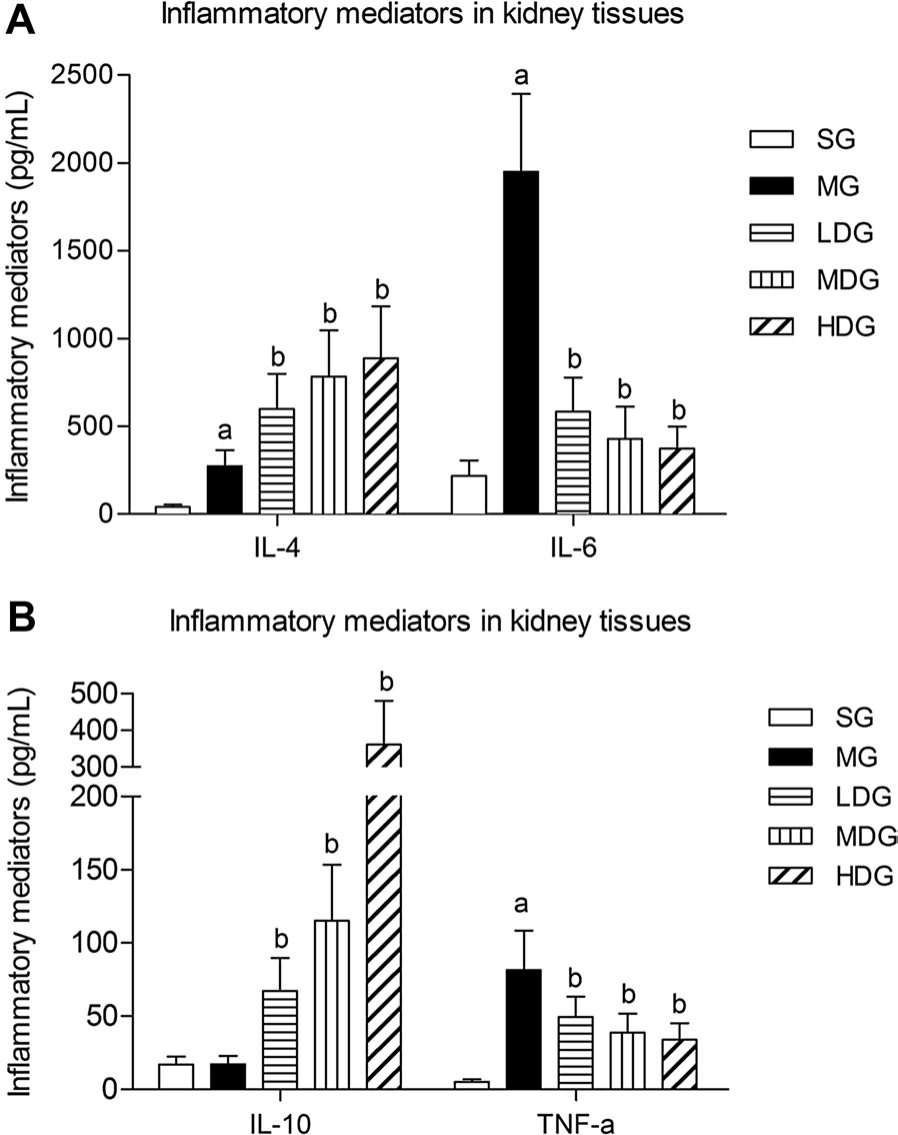
Effects of different dosages of DCQD on the inflammatory mediators in kidney tissues of rats with SAP. *SG* sham operation group, *MG* model group, *LDG* low-dose group, *MDG* medium-dose group, *HDG* high-dose group. Rats (n = 8 per group) were orally administered different doses of DCQD (6 g/kg in the LDG, 12 g/kg in the MDG, and 24 g/kg in the HDG by body weight) 2 h after operation. After 24 h, the kidney tissues were collected to determine the pro-inflammatory cytokine (IL-6 and TNF-α) and anti-inflammatory cytokine (IL-4 and IL-10) levels. The inflammatory cytokines were measured by ELISA. **A** IL-4 and IL-6 concentration in kidney tissue. **B** IL-10 and TNF-α concentration in kidney tissue. The results are expressed as the mean ± SD. ^a^*p* < 0.05 vs. SG and ^b^*p* < 0.05 vs. MG; *p* > 0.05, ns

The levels of pro-inflammatory mediators (IL-6 and TNF-α) and anti-inflammatory mediators (IL-4) in the MG were significantly increased compared to those in the SG (*p* < 0.05), but there was no change in IL-10. Compared to the MG, the pro-inflammatory mediators in all the treatment groups were downregulated (*p* < 0.05), while the anti-inflammatory mediators in all the treatment groups were upregulated obviously (*p* < 0.05). The lowest level of pro-inflammatory mediators and the highest level of anti-inflammatory mediators were both in the HDG (Fig. 2A, B).

### DCQD alleviated pathological damage in the kidney and pancreas

**Fig. 3:**
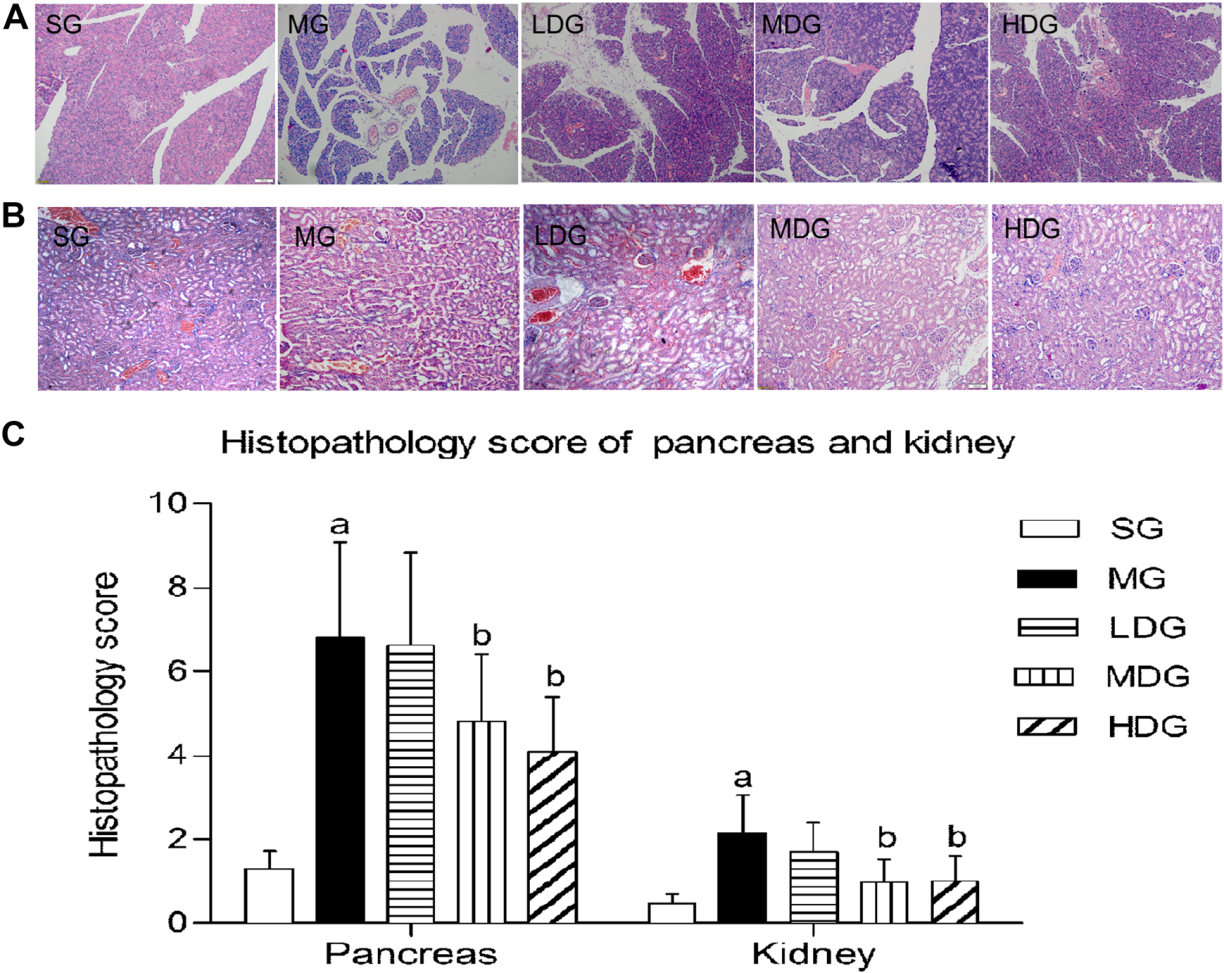
DCQD alleviates pancreas and kidney pathological damages in rats with SAP. *SG* sham operation group, *MG* model group, *LDG* low-dose group, *MDG* medium-dose group, HDG high-dose group. Rats (n = 8 per group) were orally administered different doses of DCQD (6 g/kg in the LDG, 12 g/kg in the MDG, and 24 g/kg in the HDG by body weight) 2 h after operation. At 24 h after operation, the kidney samples were collected for pathological analysis and stained with hematoxylin and eosin (HE). **A** Pathological picture of pancreas (HE, ×200). **B** Pathological picture of kidney (HE, ×100). **C** Pathological scores of the pancreas and kidney. The results are expressed as the mean ± SD. ^a^*p* < 0.05 vs. SG and ^b^*p* < 0.05 vs. MG; *p* > 0.05, ns

The pancreas of the SG rats showed slight edema, with no obvious inflammatory cell infiltration, haemorrhage or necrosis. Similar manifestations were shown in the kidneys of rats in the SG. In contrast, the pancreas in the MG displayed conspicuous interstitial edema, inflammatory cell infiltration, some spots of haemorrhage and signs of necrosis. The kidneys in the MG showed marked edema with inflammatory cell infiltration and haemorrhage. After giving DCQD, both the pancreas and kidneys in all the treatment groups had a significant reduction in interstitial edema, inflammatory cell infiltration, haemorrhage and necrosis, and the changes in the HDG were the most significant. DCQD reduced the pathological scores in the pancreas and kidneys of rats with SAP, especially in the HDG (Fig. 3A–C).

### DCQD decreased the Scr levels

**Fig. 4:**
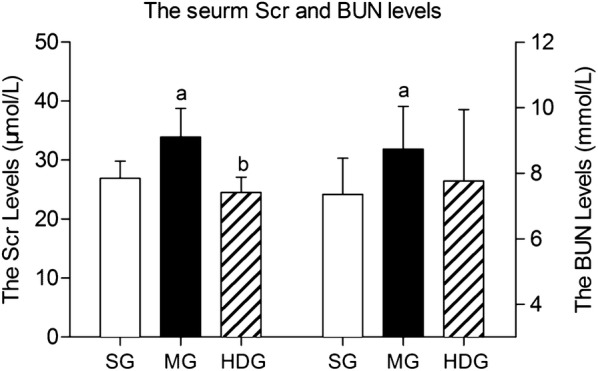
The effect of DCQD on the Scr and BUN levels in rats with SAP. *SG* sham operation group, *MG* model group, *HDG* high-dose group. Rats (n = 8 per group) were orally administered different doses of DCQD (24 g/kg in the HDG by body weight) 2 h after operation. At 24 h after operation, the blood samples were collected for the Scr and BUN analysis. The Scr levels was in the left of dotted line and the BUN levels in the right. The results are expressed as the mean ± SD. ^a^*p* < 0.05 vs. SG and ^b^*p* < 0.05 vs. MG; *p* > 0.05, ns

Based on the highest kidney distribution of DCQD and lowest kidney pathological scores in the HDG, we only detected Scr and BUN in the SG, MG and HDG. Compared with the SG, the Scr and BUN in the MG were obviously increased (*p* < 0.05). The level of Scr in the HDG was significantly lower than that in the MG (*p* < 0.05), without a difference in BUN (Fig. 4).

## Discussion

The results demonstrated that major components of DCQD were detected in the kidneys and had protective effects of regulating the inflammatory response. The distributions of major components in kidneys with SAP were similar to those in the serum, pancreas, intestine or liver [20, 22, 25]. However, there were still some differences in the kidney. The component with the highest concentration in the kidney of rats in HDG was rhein, which might be the most bioactive DCQD ingredient, even though it was less abundant than many compounds in DCQD [26], similar to that of plasma and the pancreas, emodin in lung and naringenin in intestine and liver [20, 22]. This finding once again confirms the hypothesis of the tissue pharmacology of the herbs recipe [27] and may be explained by the blood–tissue barriers in different tissues [20]. Our results showed that rhein and naringenin were relatively higher than the other compounds of DCQD in all the treatment groups. However, naringenin was the highest component in the LDG and MDG while rhein was the highest in the HDG. This phenomenon may result from the drug–drug interactions during absorption, distribution, metabolism, and excretion processes or from the procedure of decoction [28]. Zhang et al. [29] verified that Lidanpaidu prescription-a transformation of DCQD-could prevent LPS-induced AKI by restricting the NF-kB signaling pathway. Li et al. [30] proved that Huang-Lian-Jie-Du-decoction and its components had an effect on mitigating LPS-induced AKI by improving the disorder of oxidative stress and energy metabolism, preventing NF-κB and MAPK and activating the Akt/HO-1 pathway in mice. Cell research further confirmed that some single herbs were latently effective for AKI due to their ability to stop the activation of NGAL, HMGB1 and KIM-1 in an invitro AKI-mimicked condition [31]. Herein, Chinese herbs are a potentially effective treatment for AKI and are worth exploration.

AP begins with local inflammation in the pancreas, which often leads to SIRS and multiple organ failure, with high mortality [32]. The inflammatory cytokine response is initiated early and is sustained for several days in the systemic circulation during SAP [33]. The response is blamed for the systemic manifestations of AP and is related to distant organ dysfunction [34]. IL-6 is most credible in the appraisal of the severity of AP for predicting the risk for the occurrence of complications at early stages [35]. TNF-*α* can exert systemic effects on endothelial cells invivo that cause dystrophic changes to the tubular epithelial cells and cause damage to the peritubular and glomerular capillaries in the kidneys [36]. In contrast, the anti-inflammatory cytokines IL-4 and IL-10 act as potent suppressants to prevent the extracellular killing function of macrophages once activated [37]. IL-10 is likely a primary factor in the negative feedback system that hinders the production of pro-inflammatory cytokines and colony-stimulating factors in various cells [38]. Kusske et al. [39] found that IL-10 could inhibit the activation of macrophages and could alleviate inflammation by reducing the release of inflammatory cytokines and could eventually lower the death rate of SAP in mice.

In our study, we detected the levels of TNF-*α*, IL-4, IL-6, and IL-10 in kidney tissue to predict the inflammatory response after SAP modelling. The results showed that IL-6 was the highest mediators, and TNF-*α* was the lowest mediator, among those detected. Our finding echoed that IL-6 was the only significant parameter to predict a complicated AP [35]. After giving DCQD, the levels of pro-inflammatory mediators (TNF-*α* and IL-6) decreased and the anti-inflammatory mediators (IL-4 and IL-10) increased along with the dose. Moreover, the pathological scores of the kidneys and pancreas showed the same tendency. The highest dose of DCQD had the maximal effects in downregulating the pro-inflammatory mediators, upregulating anti-inflammatory mediators in the kidney and ameliorating pathological damage. The data demonstrated the regulatory effects of DCQD in the inflammatory response to ameliorate AKI with SAP and to eventually attenuate the severity of SAP. Our previous studies revealed a similar effect of DCQD on damage to the lungs, pancreas, intestine and liver [20–22]. Zhao et al. [21] reported that treatment with DCQD decreased the pathological scores of the lungs; increased the level of IL-10 mRNA and decreased the level of IL-6 mRNA in rats with SAP. Huang et al. [40] found that revised DCQD might reduce lung injury via inhibiting the induction of IL-6 and increasing the expression of HSP70 as well as the concentration of IL-10. With the experimental progress of Chinese herbs, we found that it is essential to study DCQD on the molecular level [22]. As discussed above, rhein and naringenin were relatively higher and might be potentially effective components of DCQD for SAP-induced AKI. Rhein, one major component of Dahuang, bore comparison to the accredited painkiller ibuprofen with its anti-inflammatory effects in adjuvant-induced inflammation by ameliorating oxidative stress significantly [41]. Rhein induces a necrosis-apoptosis switch of damaged pancreatic acinar cells to ameliorate AP in a dose-dependent manner [42] and to prevent an endotoxin-induced AKI by inhibiting NF-κB activities [43]. The naringenin might be another effective component and was reported to alleviate acute inflammation by adjusting the degradation by intracellular cytokines [44] and to reduce kidney damage in diabetic nephropathy through Let-7a/transforming growth factor-β1 receptor (TGFBR1) signalling [45]. Li et al. concluded that emodin had an effect on the lipopolysaccharide-induced AKI by inhibiting the toll-like receptor 2 (TLR2) signalling pathway [46]. Future studies should focus on the relationship between the quantified molecules of DCQD and their pharmacological effects considering their target tissues in SAP.

In 1998, Zhao et al. [47] came to the conclusion that the effects of DCQD on the reduction of acute phase protein levels were dose-dependent in multiple organ disfunction syndrome. Our previous study proved that the concentrations of the ten major components of DCQD increased dose-dependently in the intestine after oral administration [20]. As shown above, the effects of DCQD in the treatment of the AKI with SAP was primarily dose-dependent by regulating the inflammatory response. Therefore, a dose–response for DCQD may exist for the treatment of SAP and needs further study. The HDG accounted for the highest kidney distribution of DCQD and the lowest kidney pathological scores. Therefore, we only detected the levels of Scr and BUN in the SG, MG and HDG. Compared with the MG, the level of Scr in the HDG decreased significantly, with no differences in the BUN. Argyri et al. discovered that BUN and Scr were increased 2–3 days after AKI occurred, when 50% of the renal function was lost, and early diagnosis and intervention reduced mortality [48]. However, we collected the samples nearly one day after successful modelling. This time period might be too short to display significant changes in BUN, which could be the reason for the change in BUN after giving DCQD. Hence, it is necessary to further study and apply more sensitive biomarkers for research and clinical interpretation.

## Conclusions

In conclusion, most of the major components of DCQD were absorbed into the kidney of rats with SAP and their concentrations increased dose-dependently. Above all, DCQD ameliorated AKI in rats with SAP by regulating the inflammatory response, and it might be closely related to the intake dose.

## Additional file

**Additional file 1.** The minimum standards of reporting checklist.

## Abbreviations

DCQD: Da-Cheng-Qi-decoction
AP: acute pancreatitis
SAP: severe acute pancreatitis
SIRS: systemic inflammatory response syndrome
AKI: acute kidney injury
Scr: serum creatinine
BUN: blood urea nitrogen
HPLC–MS/MS: high-performance liquid chromatography–tandem mass spectrometry
TNF-α: tumor necrosis factor α
IL-4: interleukin-4
IL-6: interleukin-6
IL-10: interleukin-10
RRT: renal replacement therapy
