# Supplementary material for: Effect of Da-Cheng-Qi decoction for treatment of acute kidney injury in rats with severe acute pancreatitis
Source: Chin Med. 2018 Jul 13;13:38. doi: 10.1186/s13020-018-0195-8 (PMC6045888; doi:10.1186/s13020-018-0195-8)
Supplement: Supplementary file 1 — Additional file 1. The minimum standards of reporting checklist. [file 13020_2018_195_MOESM1_ESM.doc]

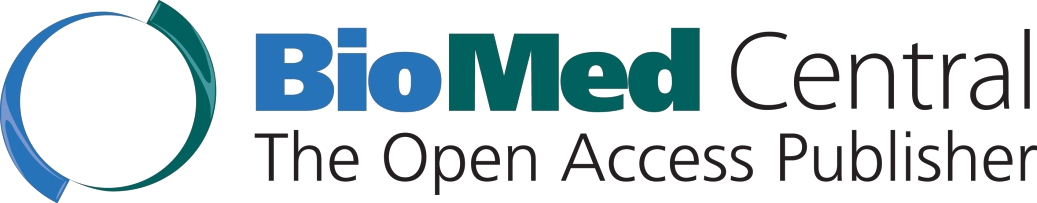


Minimum Standards of Reporting

Checklist


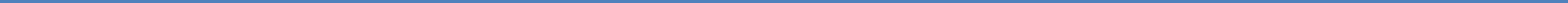


*BioMed Central* advocates full and transparent reporting. Please ensure that your paper provides the information requested below where applicable. On submitting your paper you will be asked to confirm you have included this information, or give reasons for any instances where it is not made available. You will also be asked to upload this file and it should be cited in the Methods section.

**Experimental design and statistics**

The following information should be included in the Methods section and inserted in the table below:

| **Question** | **Answer** |
| --- | --- |
| 1. The exact sample size (n) for each | Forty healthy, clean grade, male Sprague-Dawley rats (SD, 220±15 g) were purchased from Chengdu Dashuo Bio-Technique Co. Ltd. (Chengdu, China) |
| experimental group/condition (as a number, |
| not a range). Include details of a power analysis |
| if done, or any other relevant considerations |
| that determined the choice of sample size. For |
| n < 6, individual data values should be shown |
| rather than summary statistics alone. |
| 2. A description of sample collection that | Twenty-four hours after administration, all the rats were sacrificed and artery blood, kidney and pancreas tissues were collected for measurement. Pathological scores of Pancreas and kidney were evaluated. Scr and BUN were detected. The concentrations of the ten major components of DCQD in the kidney tissue homogenates (10%) were measured by high-performance liquid chromatography–tandem mass spectrometry (HPLC-MS/MS) IL-6, TNF-a, IL-4 and IL-10 in kidney tissue homogenate were measured by the Milliplex MAP Rat Cytokine/Chemokine magnetic bead immunoassay kit. |
| enables the reader to understand whether the |
| samples represent technical or biological |
| replicates, and an explanation of |
| inclusion/exclusion criteria if samples or organisms were excluded from the analysis. |
|  |
| 3. How samples/ organisms were allocated to | The rats were randomly divided into five groups and marked as the low-dose treatment group (LDG, 6 g/kg BW), the medium-dose treatment group (MDG, 12 g/kg BW), the high-dose treatment group (HDG, 24 g/kg BW), the sham-operation group (SG) and the model group (MG). |
| experimental groups and processed, and full |
| details of the randomisation procedure used (if  relevant). |
|  |
| 4. For sample assessment by human | Pathological scores were blindly evaluated by two independent pathologists with a previously established scoring system |
| investigators, a statement on whether the |
| investigator was blinded to group assignment |
| and outcome assessment, and how this blinding |
| was achieved and evaluated (if relevant). |
| 5.How many times each experiment shown was replicated and an indication of the extent of variation from experiment to experiment. | Two hours after the operation, the rats in the treatment groups were administered DCQD intragastrically at 1 mL/100 g BW, with 0.6 g/mL for the LDG, 1.2 g/mL for the MDG and 2.4 g/mL for the HDG while the rats in the SG and MG were administered an equal volume of saline. |
| 6.Information on the statistical methods and measures used. It should be clear whether the tests are one-sided or two-sided, whether there are adjustments for multiple comparisons, whether medians or means are being shown, whether error bars are standard deviations (SD), standard error of mean (SEM) or confidence intervals. | The statistical analysis was performed with PEMS3.1 for Windows (Sichuan University, China). All the data were expressed as the mean ± standard deviation (mean ± SD). One-way repeated-measures ANOVA, followed by multiple pair-wise comparisons using the Student-Neuman-Keuls procedure, was applied to the analysis of multiple groups. The data was considered significantly different when *P <*0.05. |
| 7.A justification for the appropriateness of statistical tests used to assess significance. Do the data meet the assumptions of the tests? Is there an estimate of variation within each group of data, and is the variance similar between groups that are being statistically compared?  In addition, information essential to interpreting the data presented should be made available in the figure and table legends. If the study involves health interventions for human participants, please refer to the relevant reporting guidelines from the EQUATOR Network, and the Biosharing Portal for reporting checklists for biological and biomedical research, where applicable. | The statistical analysis was performed with PEMS3.1 for Windows (Sichuan University, China). All the data were expressed as the mean ± standard deviation (mean ± SD). One-way repeated-measures ANOVA, followed by multiple pair-wise comparisons using the Student-Neuman-Keuls procedure, was applied to the analysis of multiple groups. The data was considered significantly different when *P <*0.05. |


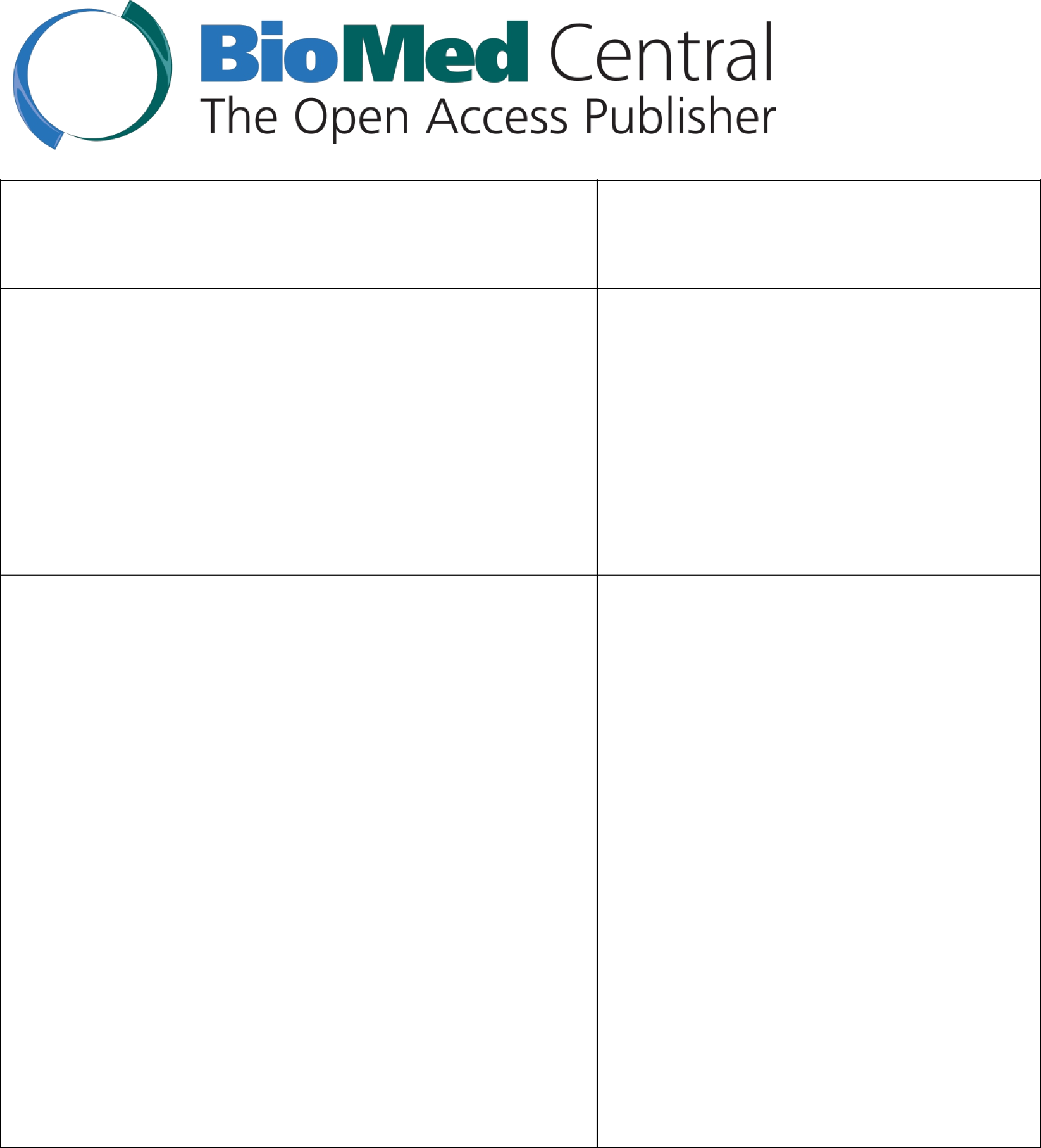


**Research involving humans**

If your research involved humans, please confirm you have adhered to the relevant reporting guideline from the [EQUATOR Network,](http://www.equator-network.org/) and included the completed checklist as an additional file with your submission:

|  | **Answer** (page and line number inserted/Not applicable for my study) |
| --- | --- |
| -I have followed the relevant reporting for my study type, and included a populated checklist with my submission  -Not applicable for my study | Not applicable for my study |


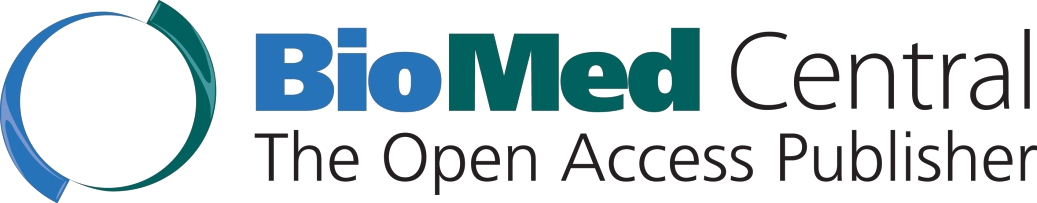


**Resources**

A description of all resources used should be included in the Methods section, with enough information to allow them to be uniquely identified. The table below should be completed with confirmation that this was done (i.e. included in the Methods section) or is not applicable. If this has not been completed, but is applicable, you should contact the journal editorial staff before proceeding.

|  | **Answer** (page and line number inserted/Not applicable for my study) |
| --- | --- |
| •Antibodies: report source, catalogue code, characteristics, dilutions and how they were validated for the system under study. | Not applicable for my study |
| •Cell lines: report source, whether identity has been authenticated and whether tested for mycoplasma contamination. We encourage researchers to check the NCBI database for contamination of cell lines. | Not applicable for my study |
| •Organisms: report source, species, strain, sex, age, husbandry, inbred and strain characteristics of transgenic and mutant animals. | page 7, line 45-59, Rat/male/SD |
| •Tools (software, databases and services): report standard tool name, provider and version number, if available. For antibodies, model organisms (mice, zebrafish and flies) and tools, authors are strongly encouraged to cite Research Resource Identifiers (RRIDs). To do so, please go to the Resource Identification Portal to search for your research resource and insert the reference text into your Methods section. | Page 11, line 25-37 |


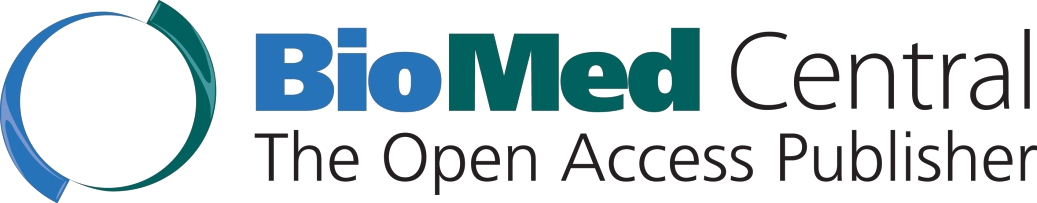


**Availability of data and materials**

The table below should be completed with confirmation that this was done (i.e. included in the Methods section) or is not applicable.

|  | **Answer** (page and line number inserted/Not applicable for my study) |
| --- | --- |
| All datasets on which the conclusions of the paper rely must be either deposited in publicly available repositories (where available and ethically appropriate) or presented in the main paper or additional supporting files, in machine-readable format whenever possible. If authors are unable to fulfill this requirement, they should contact journal editorial staff, after checking our list of Recommended Repositories. | Not applicable for my study |
| Links to deposited datasets, or datasets in additional files, should be explicitly referenced in a section entitled “Availability of Data and Materials”. Guidance on where to deposit your data can be found on the Availability of Data and Materials policy page. | Not applicable for my study |
| If computer code was used to generate results that are central to the paper’s conclusions, include a statement in the “Availability of data and materials” section to indicate how the code can be accessed. Include version information and any restrictions on availability. For deposited data and published code, a full reference with an accession number, doi or other unique identifier should be included in the reference list. | Not applicable for my study |
| If reproducible materials are generated as a result of the research (for example new animal mutants), a statement on their availability should be included. | Not applicable for my study |
